# Arbuscular mycorrhizae influence raspberry growth and soil fertility under conventional and organic fertilization

**DOI:** 10.3389/fmicb.2023.1083319

**Published:** 2023-05-16

**Authors:** Qianwen Lu, Rebecca Bunn, Erika Whitney, Yuanyuan Feng, Lisa Wasko DeVetter, Haiying Tao

**Affiliations:** ^1^Department of Plant Science and Landscape Architecture, University of Connecticut, Storrs, CT, United States; ^2^Department of Environmental Sciences, Western Washington University, Bellingham, WA, United States; ^3^Co-Innovation Center for Sustainable Forestry in Southern China, Jiangsu Province Key Laboratory of Soil and Water Conservation and Ecological Restoration, Nanjing Forestry University, Nanjing, Jiangsu, China; ^4^Northwestern Washington Research and Extension Center, Washington State University, Mount Vernon, WA, United States

**Keywords:** *Rubus idaeus* L., nutrient, fertilizer, hyphae, colonization, bioinoculum

## Abstract

**Introduction:**

Introducing beneficial soil biota such as arbuscular mycorrhizal fungi (AMF) to agricultural systems may improve plant performance and soil fertility. However, whether bioinocula species composition affects plant growth and soil fertility, and whether fertilizer source influences AMF colonization have not been well characterized. The objectives of this research were to: (1) assess if AMF bioinocula of different species compositions improve raspberry (*Rubus idaeus* L.) performance and characteristics of soil fertility and (2) evaluate the impact of fertilizer source on AMF colonization.

**Methods:**

Five bioinocula with different AMF species compositions and three fertilizer sources were applied to tissue culture raspberry transplants in a randomized complete block design with eight replicates. Plants were grown in a greenhouse for 14 weeks and plant growth, tissue nutrient concentrations, soil fertility, and AMF root colonization were measured.

**Results:**

Shoot K and Zn concentrations as well as soil pH and K concentration increased in the Commercial Mix 1 treatment (*Glomus*, *Gigaspora*, and *Paraglomus* AMF species) compared to the non-inoculated control. RFI (raspberry field bioinoculum; uncharacterized AMF and other microbiota) increased soil organic matter (SOM), estimated nitrogen release (ENR), and soil copper (Cu) concentration compared to the non-inoculated control. Furthermore, plants receiving the Mix 1 or RFI treatments, which include more AMF species, had greater AMF root colonization than the remaining treatments. Plants receiving organic fertilizer had significantly greater AMF colonization than conventionally fertilized plants.

**Conclusion:**

Taken together, our data indicate that coupling organic fertilizers and bioinocula that include diverse AMF species may enhance raspberry growth and soil fertility.

## 1. Introduction

To meet the need of feeding a growing population with limited arable land, growers must maintain or increase soil fertility to optimize crop productivity and minimize negative impacts associated with overfertilization. The addition or reintroduction of beneficial soil microbiota, such as AMF, has potential to maintain or increase soil fertility as well as plant productivity. AMF are a type of endomycorrhizae that belong to phylum Glomeromycota. These fungi establish symbiotic relationships with 70–90% of land plant species (Parniske, 2008; Genre et al., 2020). AMF form tree-shaped subcellular structures (i.e., arbuscules) within plant root cortical cells that serve as the main site of nutrient exchange between the fungus and plant symbiotic partner (Parniske, 2008). Through this symbiosis, AMF provide host plants with access to water and nutrient resources *via* their extensive extraradical hyphal network developed both inside and outside the host plant’s roots in exchange for photosynthetically derived carbon (C) (Smith and Read, 2008; Fellbaum et al., 2014).

Arbuscular mycorrhizal fungi are an important class of beneficial microorganisms that may contribute to soil health (Smith and Read, 2008). A pot experiment carried out in an open area in Faisalabad, Pakistan reported that soil polluted with lead-acid battery effluents and treated with AMF inoculum [a mixture of *Funneliformis mosseae* (T.H. Nicolson and Gerd.) C. Walker and A. Schüßler, *Rhizophagus aggregatus* (N.C. Schenck and G.S. Sm.) C. Walker, *Claroideoglomus etunicatum* C. Walker and A.Schüssler, and *Rhizophagus irregularis* (Błaszk., Wubet, Renker and Buscot) C. Walker and A. Schüßler] had greater soil microbial biomass C, total glomalin-related soil protein, phosphomonoesterase, and catalase than the non-inoculated control when grown with ‘Rakshan-10’ barley (*Hordeum vulgare* L.) for 120 days (Khan et al., 2020). In another study, a greenhouse experiment carried out in Razavi Khorasan, Iran showed that calcareous soil treated with single species AMF inoculum (*F. mosseae*) had greater soil microbial biomass carbon, dissolved organic carbon, and available iron (Fe), manganese (Mn), and zinc (Zn) concentrations relative to the non-mycorrhizal addition control (Dehghanian et al., 2018). These studies highlight that AMF can benefit soil fertility and biological characteristics, however, it is both AMF species and host plant species dependent.

Floricane red raspberry is a globally important fruit crop that is widely consumed and prized for being rich in vitamins, mineral nutrients, and dietary fiber, which are beneficial to the human body (Rao and Snyder, 2010). Raspberry growers are often challenged with the dilemma of balancing short-term goals of maximizing yields and returns on investment against practices that benefit beneficial soil microbes and overall soil health. Improvements in plant performance with the inoculation of AMF have been observed in numerous experiments. Previous studies reflect that certain plant species are generally more responsive to mycorrhizal colonization than others and responses may be both host plant and AMF specific (Rowe et al., 2007; Parniske, 2008; Chen et al., 2022). For instance, Li et al. (2017) demonstrated that *Elsholtzia splendens* Nakai ex F. Maek. seeds and seedlings inoculated with AMF (AMF in the rhizosphere soil of natively grown *E. splendens*) outperformed those without AMF inoculation for seed morphology parameters and germination rate under Cu addition at high rates (1,000 mg kg^−1^), indicating AMF can facilitate plant adaptation to Cu stress. Another study reported greater ‘Tulameen’ raspberry flower number, fruit number, and yield among plants inoculated with a single species of commercial inoculum containing AMF (*R. irregularis*) compared to non-inoculated plants in a field study in the Netherlands (Chen et al., 2022). Pot-cultured field pea (*Pisum sativum* L.) performance was also improved with the addition of mixed species AMF inoculum (*R. irregularis*, *F. mosseae*, and *R. clarum*) than the single species (*R. irregularis*) with regards to plant biomass and N and P uptake (Jin et al., 2013). However, the specific effects of AMF with different species compositions on plant performance of floricane red raspberry, which is a unique perennial fruit crop with biennial canes that are vegetative in the first year of growth and fruiting the following year before senescing, has not been studied.

Raspberry growers typically choose mineral N fertilizers, such as urea, over organic derived N fertilizers such as compost or manure (Rudolph et al., 2019). Agricultural management practices such as fertilizer choice can impact AMF performance. Studies show the response of fertilized plants to AMF bioinocula depends on the interaction between the fertilizer source and AMF. For instance, inorganic N fertilization (NaNO_3_) enhanced AMF colonized root length (colonized root length = percentage colonization × root length) of pot-grown pretransplant rice (*Oryza sativa* L.) but decreased the percentage of AMF colonization relative to the no-fertilizer control (Dhillion and Ampornpan, 1992). A field experiment carried out in Switzerland showed that the percentage of root length colonized by AMF was higher in winter wheat (*Triticum aestivum* L. cv. Sardona) grown in soils fertilized with composted farmyard manure or without fertilizers than in soils fertilized exclusively with mineral fertilizers (Mäder et al., 2000). However, the impact of fertilizer sources on AMF function has never been evaluated for a perennial woody species like raspberry.

In this study we aimed to: (1) assess if AMF bioinocula of different species compositions improve raspberry performance and characteristics of soil fertility and (2) evaluate the impact of fertilizer source on AMF colonization. We hypothesized that: (1) AMF bioinocula with diverse species compositions can enhance vegetative plant performance and characteristics of soil fertility and (2) a greater percentage of AMF root colonization will be observed with organic fertilization compared to conventional fertilization.

## 2. Materials and methods

### 2.1. Experimental design and preparation

A greenhouse experiment was conducted at the Washington State University Northwestern Washington Research and Extension Center in Mount Vernon, WA, in 2019. A factorial experiment (three fertilizer sources × five bioinocula) was established as a randomized complete block design with eight replications. The three fertilizer source treatments included: (1) conventional fertilizer [nitrogen (46-0-0), phosphate (0-45-0), and potash (0-0-60); referred to as “conventional”]; (2) organic certified liquid fertilizer derived from digested plant materials (3-2-2; referred to as “organic”); and (3) a minimal fertilizer control (referred to as “fertilizer control”). Rates of N, P, and K were determined based on a caneberry nutrient management guide (Hart et al., 2006) and were the same between conventional and organic fertilizer treatments. The five bioinocula treatments included three commercial AMF inocula (referred to as “Mix 1,” “Mix 2,” “Mix 3”), raspberry field inoculum (referred to as “RFI”), and a non-inoculated control (referred to as “AMF control”). Fertilizer and commercial AMF inocula product information are shown in Table 1.

**Table 1:** Fertilizer sources **(A)** and bioinocula products **(B)** applied to tissue culture ‘Meeker’ red raspberry plants grown in a greenhouse, WA, United States in 2019.

**(A)**		
Fertilizer product	Fertilizer analysis	Description
Nitrogen	46-0-0	Prilled nitrogen
Phosphate	0-45-0	Available phosphate P_2_O_5_
Potash	0-0-60	Available potash K_2_O
Organic	3-2-2	Liquid fertilizer derived from digested plant materials

**(B)**
Bioinocula	Mycorrhizal fungi species	Spore density (spores g inoculum^−1^)	Application method and rate
Mix 1	*Glomus aggregatum* N.C. Schenck and George S. Smith*, G. etunicatum* W.N. Becker and Gerd.*, G. clarum* Nicol. and Smith*, G. deserticola* Trappe et al.*, G. intraradices* N.C. Schenck and George S. Smith*, G. monosporus* Gerd. and Trappe*, G. mosseae* (Nicol. and Gerd.) Gerd. and Trappe*, Gigaspora margarita* W.N. Becker and I.R. Hall*, and Paraglomus brasilianum* (Blaszk.) C. Renker Blaszk. and F. Buscot	Minimum 165^z^	10 cm^3^ layered in middle of Deepot
Mix 2	*G. intraradices, G. mosseae, G. aggregatum, and G. etunicatum*	132^z^	Root dip in 0.6 g L^−1^ solution for 5 s
Mix 3	*Rhizophagus intraradices* (N.C. Schenck and G.S. Sm.) C. Walker and A. Schüssler	300^z^	10 cm^3^ layered in middle of Deepot
Raspberry farm inoculum (RFI)	Not characterized	2 ± 0.2^y^	44 cm^3^ raspberry field soil layered in middle of Deepot

^z^Spore density as reported on the product label.

^y^Whitney, 2021. Data are means ± SE (standard error; *n* = 5).

The media in which plants were grown contained a 1:1:1 mixture of sterilized raspberry field soil, sand (Sakrete; Atlanta, GA, United States), and Turface MVP soil conditioner (PROFILE Products LLC; Buffalo Grove, IL, United States). Raspberry field soil was collected at a 30 cm depth from a commercial raspberry field in Lynden, WA, United States (48°56’N, 122°32’W; elevation 24 m) in March 2019. The soil at this site was a Tromp loam, characterized as volcanic ash and loess over glacial outwash (United States Department of Agriculture, 2019). The site had no history of phytophthora root rot [*Phytophthora rubi* (W. F. Wilcox and J. M. Duncan) Manld] and root lesion nematode [*Pratylenchus penetrans* (Cobb) Filipjev and Schuurmans Stekhoven; RLN] populations were low at 0–1 RLN per 100 g dry soil (data provided by Dr. Inga Zasada, USDA-ARS nematologist). The site was bed fumigated with Telone® C-35 (63.4% 1,3-dichloropropene; 34.7% chloropicrin; Dow Agrosciences; Indianapolis, IN, United States) at 25 l ha^−1^ in June 2017 and broadcast fertilized (11-52-0) after fumigation at the rate of 145 kg N ha^−1^. Fumigation was provided by a commercial applicator (Trident Agriculture Products, Woodland, WA). A commercial planting of ‘Wake™ Haven’ raspberry was growing in the field at the time of sampling. The collected field soil was sieved through 4-mm sieve, then steam sterilized twice at 80°C for 30 min with a 24 h rest period between two steam sterilizations. After the sterilized soil cooled down, it was homogenized in a cement mixer at a 1:1:1 ratio with sand and Turface MVP. Characteristics of the field soil and final mixed soil media are listed in Supplementary Table S1. Both field soil and mixed media are beyond or within the suggested soil nutrient critical levels for pre-plant caneberry (Supplementary Table S1).

### 2.2. Plant and mycorrhizal establishment and fertilizer treatment applications

For treatment application, Deepot [D40H (diameter 6.35 cm × depth 25.4 cm), Stuewe and Sons, Inc.; Tangent, OR, USA; 656 ml volume] was filled with the mixed soil media to 2/3 (~437 cm^3^) volume. Afterwards, bioinocula treatments were applied according to label instructions and remaining mixed soil media was added at the same time as raspberry transplanting. Application methods for all bioinocula treatments are listed in Table 1. Drainage holes on the bottom of all Deepot were partially covered by clear plastic tape to minimize the holes and prevent loss of media during irrigation throughout the experiment. Experimental set-up and planting occurred in June 2019. Tissue culture ‘Meeker’ raspberry transplants (~ 15 cm tall; Northwest Plant Company; Ferndale, WA, USA) were grown individually in sanitized Deepot containing mixed soil media. Plants were placed in a greenhouse on a 12 h day/night cycle with 150–200 μmol s^−1^ m^−2^ of supplemental photosynthetically active radiation (LED Fixtures-120 Volt).

Fertilizer treatments were surface applied on 10 July and 10 September 2019 to mimic field application (Hart et al., 2006). The application rate of each fertilizer treatment is listed in Table 2. Fertilizer applications in the fertilizer control treatment ensured plants remained alive throughout the experiment, as no fertilizer input would likely manifest into plant death. Overhead irrigation occurred every day between 0900 and 1000 h with approximately 100 ml and 50 ml of water added to each Deepot in summer and fall, respectively.

**Table 2:** Application rate of different sources of fertilizers applied on 10 July and 10 September 2019 to tissue culture ‘Meeker’ red raspberry plants grown in a greenhouse, WA, United States in 2019.

Treatment	Application rate		
	N (kg ha^−1^)	P_2_O_5_ (kg ha^−1^)	K_2_O (kg ha^−1^)
Conventional	53	37	36
Organic	53	37	36
Fertilizer control	5	37	36

### 2.3. Data collection

Plant heights were recorded at harvest. Plants were harvested by removing individual juvenile plants from Deepot after 14 weeks. Roots and shoots were separated, and roots were gently rinsed in deionized water to remove soil particles. Both roots and shoots were then dried at 60°C for 48 h and weighed to determine root, shoot, and total (root + shoot) biomass. Dried raspberry shoots were sent to Brookside Laboratories, LLC (New Bremen, OH, United States) for total nutrient concentration analyses, including nitrogen (N), phosphorus (P), potassium (K), calcium (Ca), magnesium (Mg), sulfur (S), Fe, Cu, Mn, Zn, and boron (B), using methods described by Miller et al. (2013). Dried shoots were grinded through Cyclotech Mill with a 0.50 mm screen prior to the analyses. Shoot N concentration was measured by the combustion method (Elementar EL Cube C/N combustion analyzer, Elementar Inc., Langenselbold, Germany). Other shoot nutrient concentrations were measured using the nitric acid and hydrogen peroxide digestion method and then tested with inductively coupled plasma mass spectrometry (Thermo 6,500 Duo; Thermo Instruments, Waltham, MA, United States) (Miller et al., 2013). Soil from each Deepot was packed and sent to Brookside Laboratories, LLC for analysis of selected chemical characteristics: soil pH, SOM, ENR, and selected nutrient concentrations. SOM was measured using the loss of ignition method at 360°C for 12 h (Miller et al., 2013). ENR was estimated based on the percentage of organic matter in the soil. Soil P was extracted using the Bray extraction method (Miller et al., 2013) and soil K, Ca, Mg, S, Fe, Cu, Mn, Zn, and B were extracted using the Mehlich III extraction method (Mehlich, 1984) and then tested with an inductively coupled plasma spectrometer (Thermo Scientific ICAP 7000 series; Thermo Instruments, Waltham, MA, United States; Miller et al., 2013).

A random sample of 1–2 cm roots was collected from each plant and stored in uni-cassettes (Electron Microscopy Sciences Supplier Diversity Partner; Hatfield, PA, United States). Roots in uni-cassettes were prepared for AMF colonization assessment using the method outlined by Brundrett et al. (1996). One slide was prepared for each plant and 12 representative, 1-cm-stained root segments were selected per uni-cassette to make root slides for AMF colonization assessment. Slides were viewed at 200x using a compound microscope (Eclipse 50i, Nikon; Tokyo, Japan). AMF colonization was counted using the magnified intersections method (McGonigle et al., 1990) and approximately 72 intersections were assessed for each plant.

### 2.4. Statistical analyses

All analyses were performed in R (R version 3.6.0; Boston, MA, United States). We used linear mixed-effects models to determine if bioinocula type, fertilizer, or an interaction between these two factors affected final plant height, shoot, root, and total biomass, shoot nutrient concentrations, soil characteristics, or AMF colonization using the function lme() in the nlme package (Pinheiro et al., 2022). Cell means instead of main factor means will be presented when the interaction is significant. We used analysis of variance to compare each null model to the full model, “Response ~ Fertilizer*Bioinocula, random = ~1|Block” where Block was a random variable accounting for possibility that microclimates within the greenhouse affected plant height, biomass, shoot nutrient concentrations, soil characteristics, or AMF colonization. In each case, the full model explained more variance (*p* < 0.05) than the null model “Response ~1, random = ~1|Block.” In these full models, both fertilizer and bioinocula treatments were set as fixed effects and block was treated as a random effect. The fertilizer effect, bioinocula effect, and fertilizer × bioinocula interactions were tested.

The assumptions of normality and homogeneity of variance were checked by visual inspection of residual plots. When necessary, variables were transformed to meet these assumptions, including: log_10_ transformations of plant height, root biomass, shoot biomass, and shoot Zn concentration; and reciprocal transformations of shoot Fe concentration, soil P concentration, and AMF colonization. All means were back transformed and reported in original units. A Tukey’s honest significant difference test was used for *post hoc* comparisons at the 5% level of significance to compare treatment means using the function multcomp::cld() in the multcomp package (Hothorn et al., 2008).

## 3. Results

### 3.1. Total root colonization by AMF

**Figure 1:**
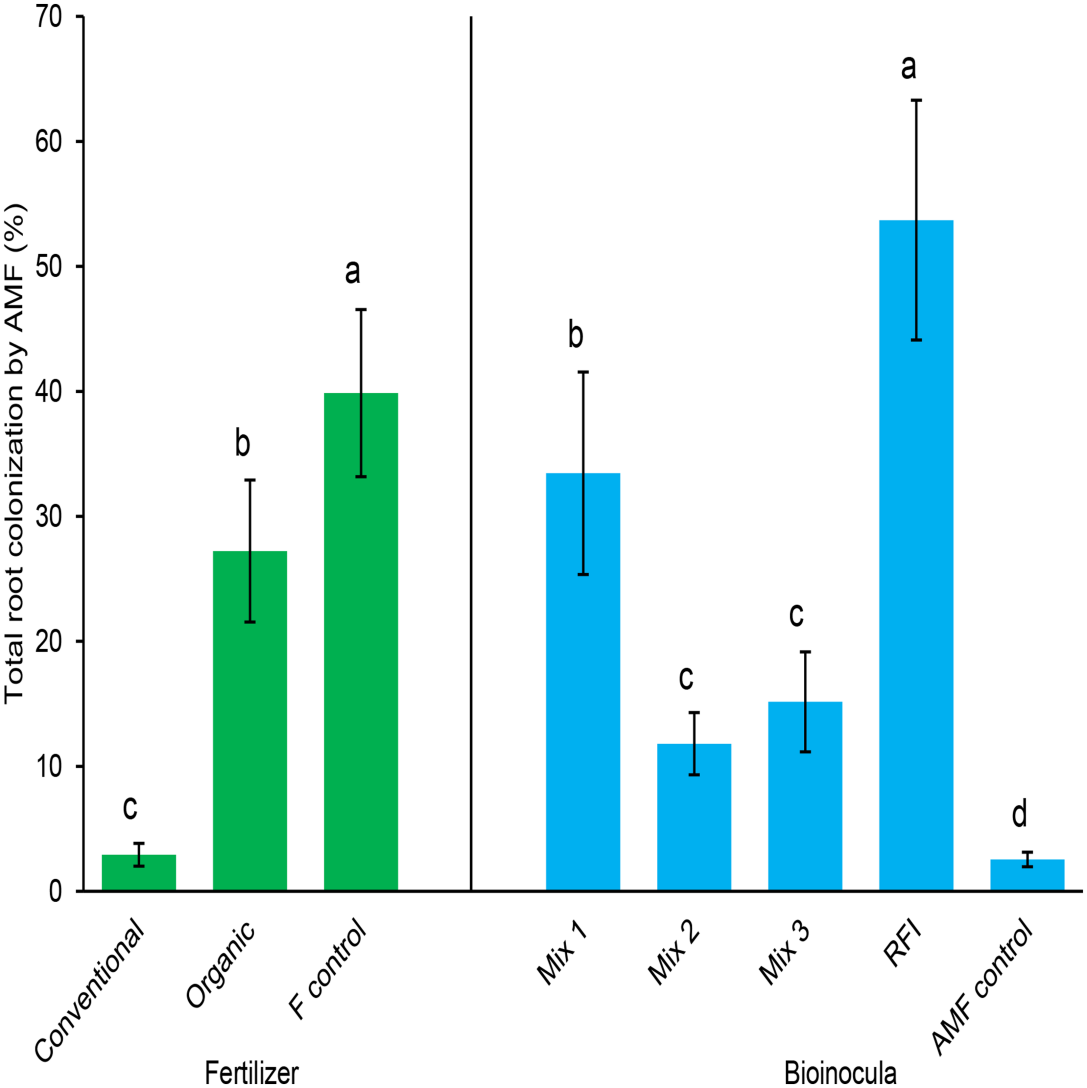
Total root colonization by arbuscular mycorrhizal fungi (AMF; %) by fertilizer **(left)** and bioinocula treatments **(right)**. Fertilizer treatments were Conventional, Organic, and F control (a minimal fertilizer control). Bioinoculant treatments were: Mix 1 (commercial AMF bioinoculum), Mix 2 (commercial AMF bioinoculum), Mix 3 (commercial AMF bioinoculum), RFI (bioinoculum collected from a commercial raspberry field), and AMF control (a non-inoculated control). Bars represent means ± SE (standard error; *n* = 20 and 12 for fertilizer and bioinocula treatments, respectively); different letters within the same treatment denotes significant differences at *p* ≤ 0.05 using a means comparison procedure with a Tukey’s honestly significant difference test.

Both fertilizer and bioinocula treatments significantly affected total root colonization by AMF (*p* < 0.0001 for both fertilizer and bioinocula treatments; Figure 1). Within fertilizer treatment, the fertilizer control resulted in the greatest total AMF colonization, followed by the organic treatment, then the conventional fertilizer treatment. Plants fertilized with conventional or organic fertilizer had 92.7 and 31.7% lower colonization, respectively, than the fertilizer control plants. Within bioinocula treatment, RFI generated the greatest total AMF root colonization, followed by Mix 1, then both Mix 2 and Mix 3 treatments, despite differences in initial spore density (Table 1). The lowest AMF root colonization was found in the AMF control treatment and neither arbuscules nor vesicles were observed, only low levels of AMF-like hyphae were found in the AMF control treatment. Mix 1 and RFI had 183 and 355% greater root colonization than Mix 2, respectively, and 121 and 254% greater root colonization than Mix 3, respectively. Root colonization by AMF in Mix 1, Mix 2, Mix 3, and RFI treatments were 1,212, 363, 495, and 2006%, respectively, greater than that in the AMF control treatment. No significant fertilizer × bioinocula interaction was detected (*p* = 0.05).

### 3.2. Plant growth variables

**Figure 2:**
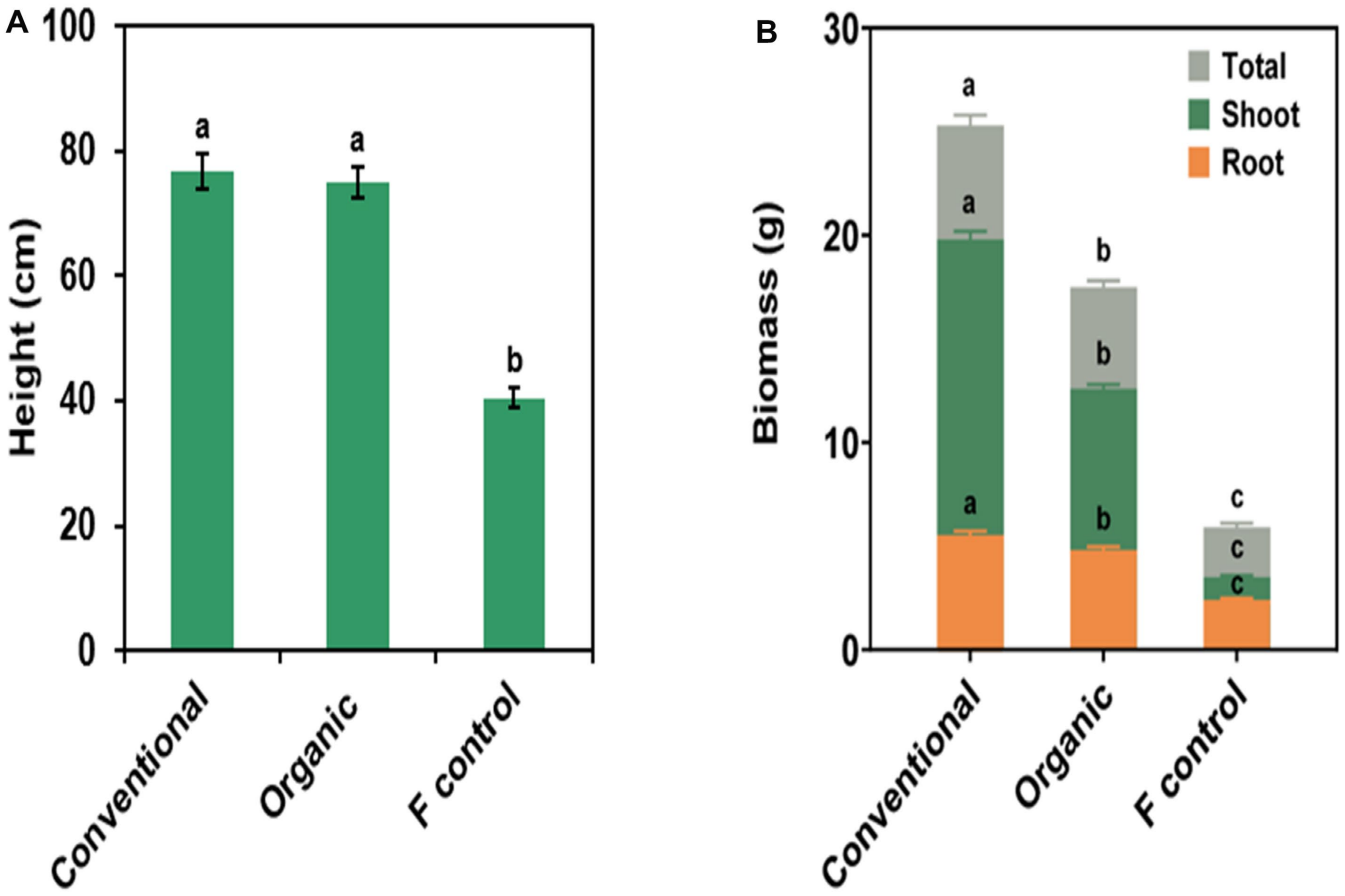
Effect of fertilizer source on **(A)** plant height, **(B)** total, shoot, and root biomass. Note total applied rates of nitrogen, phosphorus, and potassium were the same between organic and conventional fertilizer sources. Bars are represented as means ± SE (standard error; *n* = 40). In **(A,B)**, different letters on top of the bars in the same color denote a significant difference across the fertilizer source treatment at *p* ≤ 0.05 using a means comparison procedure with a Tukey’s honestly significant difference test. In **(B)**, all bars in different colors originate from the *x*-axis.

Significant fertilizer treatment effects were found in all measured plant growth variables (Figure 2 and Supplementary Table S2). Plants fertilized with conventional and organic fertilizers were significantly greater in plant height, shoot biomass, root biomass, and total biomass than the fertilizer control plants; however, there was no significant difference in plant height between plants fertilized with conventional and organic fertilizers. Shoot, root, and total biomass were significantly greater among plants treated with the conventional fertilizer, followed by plants treated with the organic fertilizer. Fertilizer control plants had the smallest shoot, root, and total biomass. All measured plant growth variables did not differ across bioinocula treatments and there were no significant fertilizer × bioinocula interactions (Supplementary Table S2).

### 3.3. Plant shoot nutrient concentrations

Shoot N and P concentrations differed significantly due to fertilizer treatment (Supplementary Table S3). Shoot N concentration was greatest in the organic fertilizer treatment, followed by the conventional fertilizer treatment, and was lowest in the fertilizer control. Shoot P concentration was significantly greater in plants fertilized with organic fertilizer than those fertilized with conventional fertilizer.

**Figure 3:**
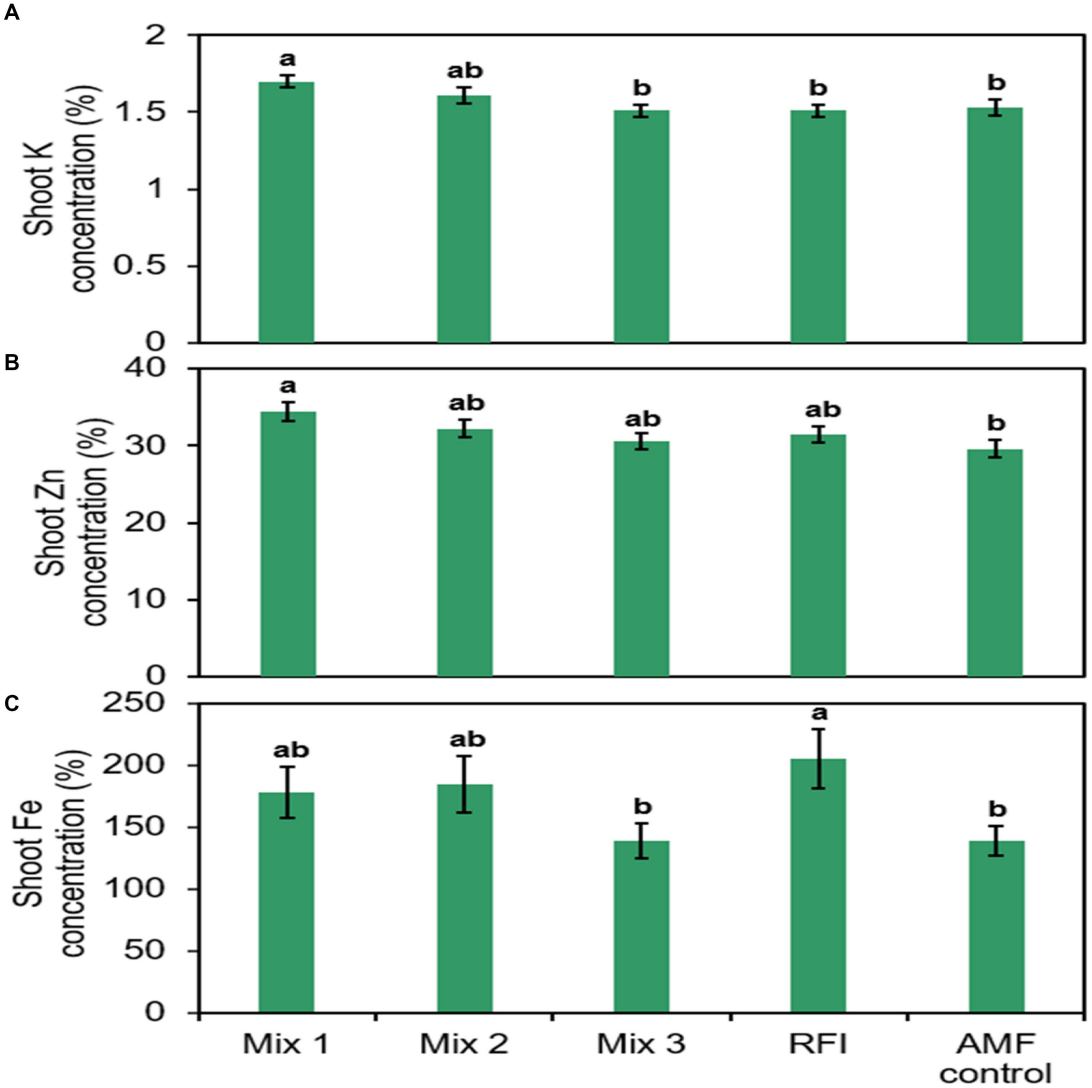
Raspberry shoot **(A)** potassium (K), **(B)** zinc (Zn), and **(C)** iron (Fe) concentrations by bioinocula treatment. Bars are represented as means ± SE (standard error; *n* = 24). Different letters within each graph on top of the bars denote a significant difference across the bioinocula treatment at *p* ≤ 0.05 using a means comparison procedure with a Tukey’s honestly significant difference test. Treatments are: Mix 1 (commercial AMF bioinoculum), Mix 2 (commercial AMF bioinoculum), Mix 3 (commercial AMF bioinoculum), RFI (bioinoculum collected from a commercial raspberry field), and AMF control (a non-inoculated control).

Shoot K, Zn, and Fe concentrations differed significantly among bioinocula treatments (Figure 3; Supplementary Table S3). Plants inoculated with Mix 1 had 12.6, 12.6, and 11.1% greater shoot K concentrations than those inoculated with Mix 3, RFI, and the AMF control, respectively. Shoot K concentration in plants inoculated with Mix 2 did not differ from plants inoculated with other bioinocula and the AMF control. Plants inoculated with the Mix 1 had greater shoot Zn concentrations than AMF control plants while shoot Zn concentrations in plants inoculated with Mix 2, Mix 3, RFI were similar to one another and the remaining treatments, including the AMF control. Plants inoculated with RFI had greater shoot Fe concentration than Mix 3. There was no significant fertilizer × bioinocula interaction for shoot N, P, K, Ca, Mg, S, Cu, Mn, Zn, and B concentrations. However, a significant interaction was found for shoot Fe concentration (Supplementary Table S3).

### 3.4. Soil fertility characteristics

**Figure 4:**
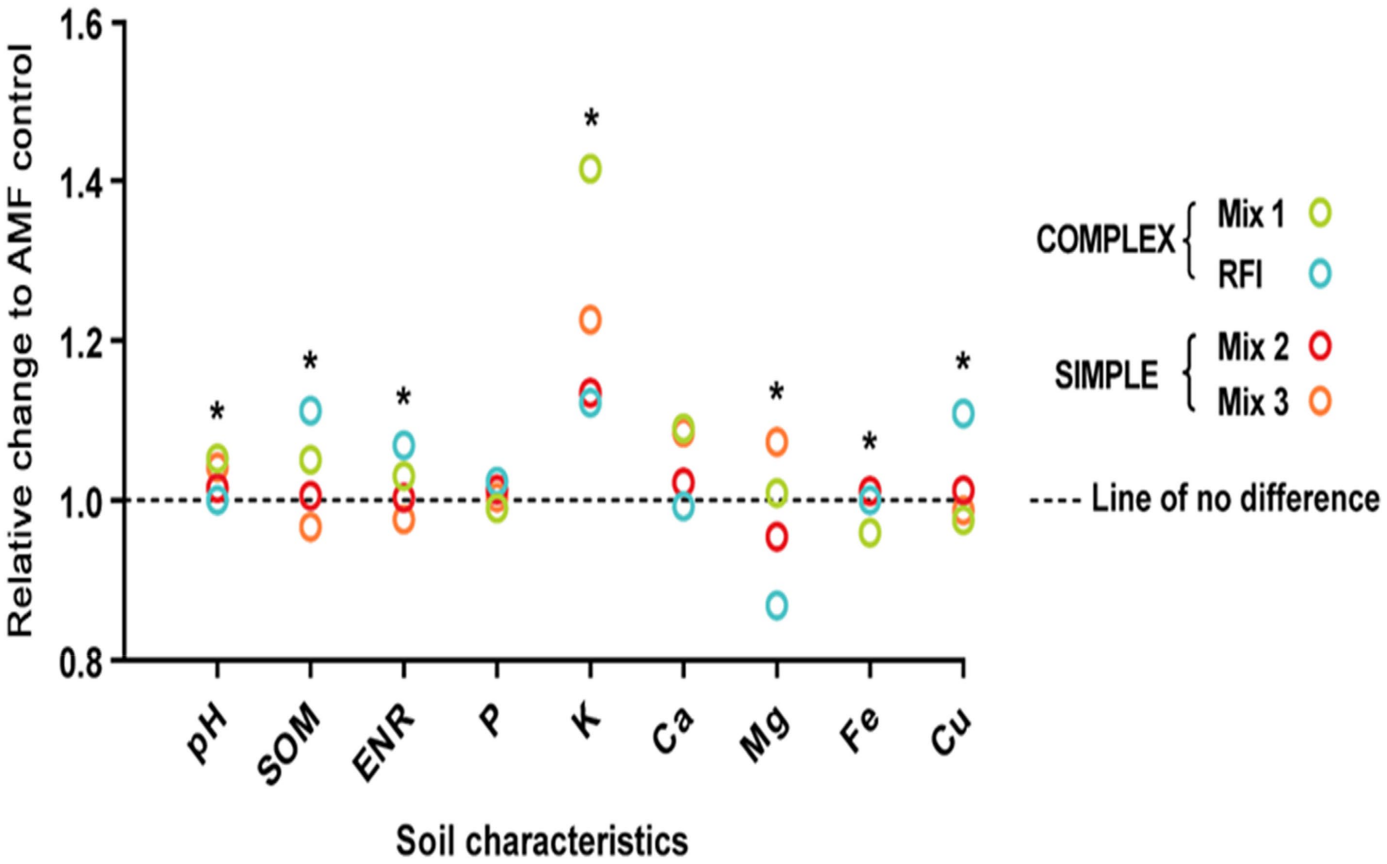
Effects of bioinocula on soil fertility characteristics expressed as relative changes of bioinocula treatments with complex [Mix 1; RFI (bioinoculum collected from a commercial raspberry field)] or simple (Mix 2; Mix 3) arbuscular mycorrhizal fungi (AMF) species diversity. The relative change in each soil fertility characteristic was calculated as the non-AMF-control treatment (Mix 1, Mix 2, Mix 3, and RFI) divided by AMF control. Asterisks within each soil characteristic represent significant differences across the bioinocula treatment at *p* ≤ 0.05 using a means comparison procedure with a Tukey’s honestly significant difference test.

Soil pH (*p* < 0.0001), SOM (*p* = 0.002), ENR (*p* = 0.002), and concentrations of K (*p* = 0.001), Mg (*p* < 0.0001), Fe (*p* = 0.0056), and Cu (*p* < 0.0001) differed significantly due to bioinocula treatment (Figure 4 and Supplementary Table S4). Effects of bioinocula on soil fertility characteristics are expressed as relative change in soil fertility characteristics in bioinocula treatments with complex (greater AMF species diversity; Mix 1; RFI) or simple (lower AMF species diversity; Mix 2; Mix 3) AMF species compositions compared to the AMF control. The relative change in each soil fertility characteristic was calculated as the ratio of each non-AMF-control treatment (Mix 1, Mix 2, Mix 3, and RFI) to AMF control. Some measured soil fertility characteristics were greater for Mix 1 and RFI than the AMF control. Mix 1 led to 5.20% greater soil pH and 41.5% greater soil K than the AMF control. RFI generated 11.2% greater SOM, 6.87% greater ENR, and 10.9% greater Cu than the AMF control. Both Mix 2 and Mix 3 had similar measured soil characteristics relative to the AMF control. However, soil Mg concentration was greater when inoculated with Mix 3 than Mix 2 or RFI and soil Fe concentration was greater when inoculated with Mix 2 than Mix 1 or Mix 3. There were no fertilizer × bioinocula treatment interactions for soil SOM, ENR, P, K, S, Mn, Zn, and B concentrations. However, significant interactions were found for soil pH and Ca, Mg, Fe, and Cu concentrations (Supplementary Table S4).

## 4. Discussion

The utilization of bioinocula with diverse AMF species under organic fertilization regime positively affected raspberry vegetative growth and soil fertility characteristics. Natural bioinocula from the commercial raspberry field also performed better than several commercial bioinocula for multiple measured variables emphasizing agriculture management practices may have a role in augmenting AMF bioinocula effects. Total root colonization differed across both fertilizer and bioinocula treatments. In the current study, total root colonization by AMF in raspberry plants was higher in the organic and fertilizer control treatments compared to the conventional fertilizer treatment. We are not the first to observe this phenomenon. Bittman et al. (2006) reported that broadcast application of liquid dairy manure had a positive effect on AMF colonization in corn (*Zea mays* L.) compared to mineral fertilization using ammonium nitrate. Similar findings were also reported by Gryndler et al. (2006), where long-term application of cattle manure increased AMF growth compared to conventional mineral fertilizers. There are several possible explanations for this observation. First, the application of conventional fertilizers increases the availability of nutrients for a short time compared to organic and fertilizer control treatments but simultaneously inhibits AMF colonization since root colonization by AMF typically increases under conditions of limited nutrient availability but decreases when nutrients are in abundance although the underlying regulatory mechanisms regarding AMF function and nutrient availability are not well studied (Parniske, 2008; Douds et al., 2017; Jeske et al., 2018; Mariotte et al., 2018). Johnson et al. (2003) reported that nutrient enrichment led to less C allocated to colonized AMF by plants because of increased nutrient availability through fertilization, which in turn reduced AMF colonization and this might be the case in the current study. Bradley et al. (2006) reported that soil grown with the grassland species, *Schizachyrium scoparium* (Michx.) Nash, had lower AMF FAME (i.e., a biomarker for AMF) abundance when fertilized with pelletized NH_4_NO_3_ than a no fertilizer control, which again showed that conventional fertilizers can decrease AMF abundance and subsequent colonization. Second, the application of urea could reduce rhizosphere pH (Monsant et al., 2008) relative to organic and control fertilization and the low rhizosphere pH might suppress AMF activity due to induced H^+^ and Al^3+^ toxicity, which can decrease extra-radical mycelium production and subsequent AMF root colonization intensity (Cruz-Paredes et al., 2021).

Different bioinocula treatments resulted in different levels of AMF root colonization and colonization was greatest in the RFI bioinoculum treatment. This was not expected because RFI bioinoculum was collected from a fumigated raspberry field that also received routine applications of conventional fertilizers in accordance with commercial production guidelines (Hart et al., 2006; DeVetter et al., 2023). Common agricultural practices (e.g., fumigation and fertilization) have long been associated with diminished species richness, abundance, and infectivity of AMF (Belay et al., 2015; Dangi et al., 2015). In the current experiment, though relatively low spore density observed in RFI than other commercial bioinoculum treatments, the application rate of RFI was relatively greater than other commercial bioinoculum treatments according to the application method (Table 1). Interestingly, Ryan and Graham (2018) demonstrated that detrimental impacts of standard agricultural practices on AMF richness, abundance, and infectivity may be limited due to the relatively low concentrations and toxicity of applied fumigants, ineffective sealant of fumigants, and poor permeability in soil due to soil texture and structure. This may be applicable to commercial raspberry system from which the RFI bioinoculum was obtained. Furthermore, the RFI bioinoculum was obtained from a field fumigated in 2017 using 1,3-dichloroprone, which has more nematocidal activity and would have been less detrimental to AMF than other commercial fumigants that contain greater concentrations of fungicides (Ibekwe et al., 2001; De Cal et al., 2005; Dangi et al., 2015). Moreover, the two-year interval between soil fumigation and sampling might have allowed soil fungi like AMF to repopulate (Brady and Weil, 2015; Rudolph et al., 2019). An additional possible explanation for greater AMF colonization observed in the RFI bioinoculum treatment is that the reduction in AMF diversity and abundance caused by commercial agricultural practices could be replenished *via* the transmission of AMF from adjacent fields and semi-natural habitats by animals or wind (Warner et al., 1987; Allen et al., 1989; Vernes and Dunn, 2009; Lekberg et al., 2011). Another possibility is that the AMF community in the RFI bioinoculum treatment has adapted to the management practices applied to the soils used in this study, thus colonization was less inhibited (Pellegrino et al., 2019). Further research should explore re-colonization mechanisms to inform how to manage soils that could be biologically compromised through standard agricultural practices like soil fumigation.

Reduced AMF root colonization in Mix 2 and Mix 3 compared to Mix 1 and RFI treatments may be due to lower AMF species diversity in Mix 2 and Mix 3. Mix 1 contained nine AMF species and RFI contained a wide range of soil microorganisms whereas Mix 2 only has four AMF species and Mix 3 is a single species product (Table 1). These findings in the current study align with previous work that found late-successional plant species tend to have greater root colonization with locally collected field inoculum and with commercial AMF inocula that contained higher species diversity compared with those containing a single species in a greenhouse study (Rowe et al., 2007). Greater root colonization by AMF might be associated with the greater species diversity in locally collected field inoculum and commercial AMF inoculum containing high species diversity (Mirmajlessi et al., 2018).

Plants receiving conventional fertilizer had greater shoot biomass than those treated with organic fertilizer whereas shoot N and P concentrations were greater in the organic fertilizer than in the conventional fertilizer treatments. This response was attributed to a ‘dilution effect’ as both treatments had the same N and P rate, but differed in shoot biomass (Rempel et al., 2004; Zhou and Yin, 2018). Shoot N and P concentrations were below the documented sufficiency ranges but shoot K, Ca, Fe, and Zn concentrations were within or above the documented sufficiency range of these nutrients (2.3–3.0% for N, 0.19–0.45% for P, 1.3–2.0% for K, 0.6–2.0% for Ca, 60–250 ppm for Fe, 15–50 ppm for Zn) recommended by Strik (2013). Although a significant effect of Mix 1 relative to the AMF control was observed for increasing shoot K and Zn concentrations, whether the small increase in shoot nutrient concentrations could in turn enhance plant productivity remains questionable. Furthermore, nutrient sufficiency guidelines are based upon samples being collected from newly expanded field-grown primocane leaves between late-July and early-Aug (Strik, 2013), whereas our sampling time was late-October and the whole above-ground plant shoot was subject to nutrient analyses due to the nature and maturity of our samples. Regardless, results in our study mirror previous work that showed inoculation by AMF increased shoot K and Zn concentrations of *Crotalaria juncea* L. grown in a soil with high Cu concentrations and increased shoot Zn concentration of maize (*Zea mays* L.) grown in a calcareous soil compared to the non-inoculated controls (Ademar Avelar Ferreira et al., 2015; Dehghanian et al., 2018).

It is interesting to note that plants inoculated with Mix 1 had significantly greater shoot K concentrations than plants inoculated with Mix 3 or RFI, however, a growth effect was not found given plants had similar height and biomass across bioinocula treatments in the current study. This finding indicates that it might take time to reflect the positive effect of AMF inoculation on plant growth and productivity. However, plants are known to engage in luxury uptake of K so growth responses are expected to be nutrient dependent (Kang et al., 2014). The greater shoot K concentration found in plants treated with Mix 1 than Mix 3 may be due to greater AMF colonization and species diversity of the bioinocula, as AMF root colonization was greater when treated with Mix 1 (nine AMF species) compared to Mix 3 (a single species) (Table 1). These observations were consistent with previous findings showing that AMF inocula sourced from a local field, reference ecosystem, and commercial inoculum containing high AMF species diversity resulted in greater root colonization by AMF than single species inoculum (Rowe et al., 2007; Maltz and Treseder, 2015). With regards to the greater shoot K concentrations observed in plants treated with Mix 1 than the RFI bioinoculum treatment, this might be due to differences in the quality of the symbioses between the host plant and AMF (Parniske, 2008; Fellbaum et al., 2014; Bender et al., 2016).

Mix 1 has the potential to increase crop value by enhancing shoot Zn concentration as Zn is associated with improved crop production and human health (Cakmak et al., 2017). The improved nutrient status arising from a mycorrhizal association has indirectly or directly accounted for subsequent increases in plant growth (Dhillion and Ampornpan, 1992), which highlights the potential role of AMF inocula on plant growth and productivity. In addition to nutrient acquisition, AMF may help host plants against various biotic and abiotic stresses by assisting in the up-regulation of tolerance mechanisms and preventing the down-regulation of key metabolic pathways (Begum et al., 2019), aid host plant development of an enhanced defensive capacity through ‘mycorrhiza-induced resistance’ (Cameron et al., 2013), and regulate global C cycling as up to 20% of the photosynthetic products of terrestrial plants are consumed by AMF (Parniske, 2008).

The mixed soil medium used in this study had nutrient concentrations either above or within the suggested critical levels for pre-planting of caneberry (20–40 ppm for P, 150–350 ppm for K, 1000 ppm for Ca, 120 ppm for Mg, 20–60 ppm for Mn, 0.5–1.0 ppm for B) according to Strik (2013). The greater SOM content and ENR in soil media treated with RFI compared to Mix 2, Mix 3, and the AMF control may be partially explained by the greater SOM content (3.76%; Supplementary Table S1) in the RFI, which was field soil collected from a commercial raspberry farm. The added RFI treatment increased SOM by 0.12%. Previous research asserted that RFI could contain a diverse assembly of soil microorganisms (i.e., fungi, bacteria, protozoa, etc.,) compared to other AMF treatments in this experiment (Mirmajlessi et al., 2018), which likely reproduced during the experimental period and could have resulted in greater SOM content by increasing microbial biomass (Lehmann and Kleber, 2015; Hestrin et al., 2019). Another study found that calcareous soil inoculated with AMF had greater microbial biomass C and dissolved organic C compared to the control lacking AMF (Dehghanian et al., 2018).

Greater soil pH in Mix 1 than Mix 2, RFI, and the AMF control at the end of the experiment reflected the potential of Mix 1 to resist changes in soil pH relative to other AMF treatments given the initial soil pH of the mixed soil media was 7.5. Greater soil K concentrations in Mix 1 compared to other AMF bioinocula treatments may be explained by the diverse AMF species in Mix 1 compared to other commercial AMF sources, and some of the specific species in Mix 1 may be highly efficient in decomposing and solubilizing K sources in soil and in turn enhance soil K concentrations (Rowe et al., 2007; Veresoglou and Rillig, 2012; Brady and Weil, 2015; Emam, 2016;). The greater soil Cu concentrations observed in the RFI treatment might be explained by the composition of the different microorganisms in RFI than in other AMF treatments as only the RFI treatment contained non-AMF microorganisms, such as bacteria, which can synergize with AMF to increase soil mineral nutrient concentrations (Hestrin et al., 2019; Jiang et al., 2021). For instance, it was reported that bacteria can swim in a water film along AMF hyphae, being nourished by hyphal exudates on their way toward the phytate patch, where they can cooperate with AMF to efficiently utilize inaccessible nutrient sources to release plant available nutrients (Jiang et al., 2021). In addition, the RFI treatment collected from a commercial raspberry farm might contain more diverse AMF species than other AMF treatments and some of the specific species might be highly efficient in decomposing and solubilizing Cu sources in soil and in turn enhance soil Cu concentrations (Rúa et al., 2016; Mirmajlessi et al., 2018). Similarly, Dehghanian et al. (2018) found that calcareous soil inoculated with AMF had greater Fe, Mn, and Zn concentrations than the non-inoculated control when cultivated with maize. RFI and Mix 1, both of which contained wide AMF species diversity, enhanced soil fertility by increasing SOM content and soil K and Cu concentrations compared to other AMF bioinocula treatments and have the potential to promote plant productivity and soil health in the commercial production system.

## 5. Conclusion

Total root colonization by AMF was greatest when plants were inoculated with bioinoculum RFI followed by Mix 1 which had high species richness relative to the other evaluated bioinocula. AMF root colonization was also influenced by fertilizer sources with AMF root colonization greatest in the fertilizer control followed by the organic fertilizer treatment then the conventional fertilizer treatment. Despite colonization differences, bioinocula treatments had no effect on plant performance. In contrast, plants responded to fertilizer source treatment and those receiving conventional or organic fertilizers had greater height and biomass than fertilizer control plants. Elevated levels of shoot K and Zn concentrations and soil pH and K concentration were found in plants inoculated with Mix 1 while RFI inoculum enhanced SOM, ENR, and soil Cu. These results support our hypotheses that: (1) AMF bioinocula with diverse species compositions can enhance plant performance and characteristics of soil fertility and (2) a greater percentage of root colonization by AMF will be observed with organic fertilization compared to conventional fertilization. However, greenhouse study results may differ from the field and subsequent studies should be conducted in the field to validate our findings. Results from the study will allow researchers, crop consultants, and growers to have a sense of the potential application of commercial AMF bioinocula.

## Data availability statement

The original contributions presented in the study are included in the article/Supplementary material, further inquiries can be directed to the corresponding authors.

## Author contributions

QL: conceptualization, methodology, software, validation, formal analysis, investigation, data curation, writing–original draft and review and editing, and visualization. RB: conceptualization, methodology, resources, writing–review and editing, and funding acquisition. EW: methodology. YF: resources and writing – review and editing. HT: resources, writing – review and editing, and funding acquisition. LD: conceptualization, methodology, resources, writing–review and editing, project administration, and funding acquisition. All authors contributed to the article and approved the submitted version.

## Funding

We gratefully acknowledge the financial support from the Washington State Department of Agriculture (WSDA) through the United States Department of Agriculture (USDA) Specialty Crop Block Grant (SCBG) (No. K2299) and Washington Red Raspberry Commission (WRRC) (No. 2019 WRRC Bunn/DeVetter). This project was also supported by the USDA National Institute of Food and Agriculture Hatch projects 1014919 and 1014527, and the China Scholarship Council (No. 201808320400).

## Conflict of interest

The authors declare that the research was conducted in the absence of any commercial or financial relationships that could be construed as a potential conflict of interest.

## Publisher’s note

All claims expressed in this article are solely those of the authors and do not necessarily represent those of their affiliated organizations, or those of the publisher, the editors and the reviewers. Any product that may be evaluated in this article, or claim that may be made by its manufacturer, is not guaranteed or endorsed by the publisher.
