# Supplementary material for: Arbuscular mycorrhizae influence raspberry growth and soil fertility under conventional and organic fertilization
Source: Front Microbiol. 2023 May 16;14:1083319. doi: 10.3389/fmicb.2023.1083319 (PMC10227501; doi:10.3389/fmicb.2023.1083319)
Supplement: Supplementary file 1 [file Table_1.docx]

**Table S1.** Characteristics of field soil and mixed soil media comprised of sterilized field soil, sand, and turface MVP at a 1:1:1 ratio. The mixed soil media treated with different fertilizer and bioinocula treatments was used in the greenhouse experiment for growing tissue culture ‘Meeker’ floricane red raspberry in 2019.

|  | pH^z^ | Organic matter (%) | mg kg^-1^ | | | | | | | | | | | |
| --- | --- | --- | --- | --- | --- | --- | --- | --- | --- | --- | --- | --- | --- | --- |
|  |  |  | NO_3_-N | NH_4_-N | P^y^ | K | Ca | Mg | S | Mn | B | Fe | Cu | Zn |
| Field soil | 7.6 | 3.76 | 6.5 | 4.2 | 300 | 247 | 2792 | 130 | 13 | 33 | 0.62 | 176 | 6.31 | 6.66 |
| Mixed soil media | 7.5 | 1.85 | 1.0 | 4.6 | 313 | 206 | 1516 | 213 | 48 | 49 | 1.00 | 244 | 4.41 | 5.13 |
| Critical level^x^ | 5.6–6.5 | N/A^w^ | N/A | N/A | 20–40 | 150–350 | 1000 | 120 | N/A | 20–60 | 0.5–1.0 | N/A | N/A | N/A |

^z^ Measured using soil:H_2_O at a 1:1 ratio.

^y^ Bray P.

^x^ Suggested soil nutrient critical levels for pre-plant caneberry (Strik, 2013).

^w^ Not provided.

**Table S2.** Significance (expressed as *P*-values from ANOVA) of plant height, shoot biomass, root biomass, and total biomass of 14-week-old ‘Meeker’ floricane red raspberry grown in mixed soil media treated with different fertilizer and bioinocula in 2019.

| Significance | Height (cm) | Shoot biomass  (g plant-1) | Root biomass  (g plant^-1^) | Total biomass  (g plant^-1^) |
| --- | --- | --- | --- | --- |
| Fertilizer (A) | <0.0001 | <0.0001 | <0.0001 | <0.0001 |
| Bioinocula (B) | 0.78 | 0.14 | 0.44 | 0.53 |
| Interaction (A × B) | 0.19 | 0.35 | 0.07 | 0.30 |

**Table S3.** Significance (expressed as *P*-values from ANOVA) of measured shoot nutrient concentrations of ‘Meeker’ floricane red raspberry grown in mixed soil media treated with different fertilizer and bioinocula in 2019.

| Significance | % | | | | | |  | mg kg^-1^ | | | | |
| --- | --- | --- | --- | --- | --- | --- | --- | --- | --- | --- | --- | --- |
|  | N | P | K | Ca | Mg | S |  | Fe | Cu | Mn | Zn | B |
| Fertilizer (A) | <0.0001 | 0.03 | 0.33 | <0.0001 | 0.004 | <0.0001 |  | <0.0001 | 0.14 | <0.0001 | 0.01 | <0.0001 |
| Bioinocula (B) | 0.60 | 0.44 | 0.01 | 0.10 | 0.03 | 0.61 |  | 0.02 | 0.07 | 0.40 | 0.04 | 0.64 |
| Interaction (A × B) | 0.17 | 0.18 | 0.89 | 0.75 | 0.93 | 0.05 |  | 0.02 | 0.19 | 0.12 | 0.07 | 0.34 |

**Table S4.** Significance (expressed as *P*-values from ANOVA) of measured soil characteristics of mixed soil media used for growing ‘Meeker’ floricane red raspberry treated with different fertilizer and bioinocula. Soil samples were tested after the harvest of raspberry plants, 2019.

| Significance | pH | SOM | ENR | mg kg^-1^ | | | | | | | | | |
| --- | --- | --- | --- | --- | --- | --- | --- | --- | --- | --- | --- | --- | --- |
|  |  | (%) | (kg N ha^-1^) | P | K | Ca | Mg | S | Fe | Cu | Mn | Zn | B |
| Fertilizer (A) | <0.0001 | 0.32 | 0.26 | <0.0001 | <0.0001 | 0.007 | <0.0001 | <0.0001 | 0.08 | 0.002 | <0.0001 | <0.0001 | 0.29 |
| Bioinocula (B) | <0.0001 | 0.002 | 0.002 | 0.83 | 0.001 | 0.05 | <0.0001 | 0.11 | 0.006 | <0.0001 | 0.41 | 0.57 | 0.11 |
| Interaction (A × B) | 0.02 | 0.88 | 0.91 | 0.14 | 0.08 | 0.02 | <0.0001 | 0.76 | 0.03 | 0.002 | 0.05 | 0.12 | 0.08 |
